# Microbiota of maize kernels as influenced by *Aspergillus flavus* infection in susceptible and resistant inbreds

**DOI:** 10.3389/fmicb.2023.1291284

**Published:** 2023-11-06

**Authors:** Geromy G. Moore, Subbaiah Chalivendra, Brian M. Mack, Matthew K. Gilbert, Jeffrey W. Cary, Kanniah Rajasekaran

**Affiliations:** ^1^Southern Regional Research Center, USDA-ARS, New Orleans, LA, United States; ^2^Department of Plant Pathology and Crop Physiology, College of Agriculture, Louisiana State University, Baton Rouge, LA, United States

**Keywords:** *Aspergillus flavus*, corn microbiome, biocontrol, bacteriome, mycobiome

## Abstract

**Background:**

Nearly everything on Earth harbors a microbiome. A microbiome is a community of microbes (bacteria, fungi, and viruses) with potential to form complex networks that involve mutualistic and antagonistic interactions. Resident microbiota on/in an organism are determined by the external environment, both biotic and abiotic, and the intrinsic adaptability of each organism. Although the maize microbiome has been characterized, community changes that result from the application of fungal biocontrol strains, such as non-aflatoxigenic *Aspergillus flavus*, have not.

**Methods:**

We silk channel inoculated field-grown maize separately with a non-aflatoxigenic biocontrol strain (K49), a highly toxigenic strain (Tox4), and a combination of both *A. flavus* strains. Two maize inbreds were treated, *A. flavus*-susceptible B73 and *A. flavus*-resistant CML322. We then assessed the impacts of *A. flavus* introduction on the epibiota and endobiota of their maize kernels.

**Results:**

We found that the native microbial communities were significantly affected, irrespective of genotype or sampled tissue. Overall, bacteriomes exhibited greater diversity of genera than mycobiomes. The abundance of certain genera was unchanged by treatment, including genera of bacteria (e.g., *Enterobacter*, *Pantoea*) and fungi (e.g., *Sarocladium*, *Meyerozyma*) that are known to be beneficial, antagonistic, or both on plant growth and health.

**Conclusion:**

Beneficial microbes like *Sarocladium* that responded well to *A. flavus* biocontrol strains are expected to enhance biocontrol efficacy, while also displacing/antagonizing harmful microbes.

## 1. Introduction

Maize (*Zea mays* L.) is an important agricultural commodity with an estimated global export value in the billions of dollars (FAS, 2021). This crop is susceptible to a variety of fungal pathogens, but the most serious is *Aspergillus flavus* due to its potential to produce the potent hepatocarcinogen known as aflatoxin (Klich, 2007; Xu et al., 2020). This ubiquitous microfungus is weakly pathogenic and therefore benefits from suppression of host defenses from external factors, such as drought stress (Scheidegger and Payne, 2003; Yu et al., 2008), to successfully infect the crop. It targets the oil-rich maize kernels, which mature from ovules connected to pollinated silks (Nielsen, 2016), suggesting the silk channel is a route the fungus can take to infect the crop (Marsh and Payne, 1984; Payne et al., 1988; Thompson and Raizada, 2018). Contamination of maize with aflatoxin is an economic issue for some of the regions where it is grown. It is also a public health issue, especially in regions that experience food scarcity or have no regulations in place for acceptable intake limits (Wild et al., 2015). Research to improve maize resistance to *A. flavus* and other aflatoxin producing aspergilli is ongoing (Rajasekaran et al., 2008; Warburton et al., 2013; Womack et al., 2020), but the most popular strategy currently is the pre-harvest use of naturally occurring, non-aflatoxigenic *A. flavus* strains as biocontrol. Proven as a successful method to reduce field aflatoxin levels for decades, formulations comprised of one or more of these strains are being developed and used all over the world (Dorner et al., 1992; Cotty, 1994; Abbas et al., 2006; Atehnkeng et al., 2014; Camiletti et al., 2018; Mauro et al., 2018; Savić et al., 2020).

One overlooked, and very important, facet of *A. flavus* biocontrol is its impact on the greater microbial communities present on or around the crop being treated. The non-aflatoxigenic strains used as biocontrol are considered more effective when they are native to the regions where they are to be used (Grubisha and Cotty, 2015). The natural field load of microbes is part of a niche balance under ordinary conditions (Dini-Andreote et al., 2015). Inundation of fields with copious amounts of the select strain(s) surely alters that balance, even if only temporarily. Therefore, it is important to determine the effects of applied biocontrol on rhizosphere and crop microbiota. By profiling the microbiomes of maize (both in the phyllosphere and endosphere), as well as the below and the above ground environment, it is possible to build a holistic and synthetic microbial community that can be used to improve crop health and yield (Singh and Goodwin, 2022). Investigating the response of each microbiome to the introduction of biocontrol may allow for the discovery of beneficial microbes that respond positively to non-aflatoxigenic *A. flavus* and form mutualistic interaction networks (Massart et al., 2015). Microbial mutualists that enhance the efficacy of *A. flavus* biocontrol could then be applied to fields, either as a probiotic ahead of biocontrol strain application, or as an additive/synergistic co-inoculant.

Microbiome studies have characterized the microbial communities associated with maize, but most focused attention on bacteriomes (bacterial communities) of the rhizosphere where there is likely to be greater diversity due to increased nutrient richness and fewer environmental stressors (both abiotic and biotic; Vorholt, 2012). The phyllosphere is considered to have lower nutrient richness and greater potential for exposure to environmental stressors, as well as greater abundance of fungal communities that contain pathogenic species (Singh and Goodwin, 2022). Our study investigated the microbial communities present on maize kernels, and further, community changes after inoculating the silks of field-grown plants with a highly toxigenic *A. flavus* strain (mimicking natural pre-harvest infection), a non-aflatoxigenic strain (mimicking pre-harvest biocontrol treatment), or a mixture of both. Each set of treatments was applied to a maize inbred that is highly susceptible to *A. flavus* infection and another that is highly resistant. Metagenomic sequencing and analysis were performed to identify microbial communities associated with maize ovule tissues. The impacts of applied treatments on these communities, and the identification of the microbes interacting with of *A. flavus* biocontrol strains, will offer better insights into biocontrol dynamics.

## 2. Materials and methods

### 2.1. Field preparation

The field study was conducted at the LSU AgCenter Doyle Chambers Research Station in Baton Rouge, LA (30.361644 N, −91.169061 W) during April–August 2018. The experimental area was treated with an herbicide mix (Bifenthrin, Atrazine, Metolachlor and Glyphosate) 1 month before planting. In accordance with APHIS regulations, test plots were isolated from other maize plots since two transgenic *A. flavus* strains were used (described in section 2.2). CML322, an inbred resistant to *Aspergillus flavus* (Betrán et al., 2002) and B73, a susceptible inbred (Campbell and White, 1995; Chalivendra et al., 2020), were planted in three replicate plots. Each plot had four 4 m rows with an interrow distance of 80 cm and interplant distance of 8–10 cm. To keep the insect pressure low, a broad-spectrum foliar insecticide known as Besiege (Syngenta Corporation, Greensboro, NC, United States) was sprayed at about the V9 (May 28th) and R1 (June 22nd) developmental stages (Abendroth et al., 2011). Inoculated B73 and CML322 inbred plants were bordered by rows of an early-maturing Bt-hybrid (Dekalb DKC67-720). This hybrid produced tassels 2–3 weeks before the experimental inbreds, which we manually detasseled to prevent pollen contamination. Since the hybrid plants were much taller than the inbred genotypes, they also acted as a shield against any stray pollen. All operations including residue disposal were carried out according to APHIS guidelines.

### 2.2. Fungal treatments

Two transgenic *A. flavus* strains were used for this study, both of which have been accessioned into the culture collection at the Southern Regional Research Center (SRRC) in New Orleans, Louisiana, United States. The wild-type biocontrol strain, K49 (SRRC 1587), was originally isolated in Mississippi and has proven successful at mitigating aflatoxin levels in multiple studies (Abbas et al., 2006, 2017). Strain K49 was one of several non-aflatoxigenic strains transformed with enhanced green fluorescent protein (eGFP) as a potential biomarker to track movement of biocontrol strains (Moore et al., 2013). It was developed as described in Rajasekaran et al. (2008) for a different *A. flavus* strain. The eGFP construct was also shown to be a heritable biomarker in *A. flavus* mating studies involving K49-GFP as a fluorescent parent (Moore, 2015). In this study, K49-GFP (SRRC 1587-G) was intended to precisely quantify its abundance in our experimental material. The wild-type toxigenic strain, Tox4 (SRRC 573; 07-C-1-1-1; LA2) was first isolated in Louisiana (Sweany et al., 2011) and considered to be appropriate for our studies since it is locally adapted. It produces both aflatoxin and cyclopiazonic acid (CPA) at concentrations up to the tens-of-thousands ppb (Moore et al., 2019, 2021, 2022). It was transformed with mCherry RFP to generate transgenic strain SRRC 573-R and distinguish it among amplicons derived from other prevailing *A. flavus* strains in our experimental plots. Both fluorescent strains grow just as well as their respective wild types. To prepare the inoculum, both strains were grown on PDA plates for 7 days at 30°C. Conidia were collected by rinsing colonies with 0.01% Triton X-100 to dislodge the spores. Resulting 500 mL spore suspensions had concentrations of 10^8^/mL in 0.001% Triton X-100.

Three days after insecticide application, the spore suspensions containing either strain or their equal mix were used to infect via silk channel injections with tree-marking guns as described before (Windham and Williams, 2012; Chalivendra et al., 2018). An additional inoculation of the emerged silks was performed by surface sprays. The control treatment had no conidia present, being solely a Triton X-100 solution.

### 2.3. Microbial DNA extraction and sequencing

Fifty days post-inoculation (R5-R6 stages), ears were harvested and placed in sterile collection pouches, placed on ice, and taken to a BSL-2 lab at LSU immediately for processing. Husks were removed, and the cobs were sliced into 5–6 cm pieces in a sterile hood. The kernel epibiota were captured by placing 10 g of ear slices in a flask containing 50 mL of sterile buffered peptone water (BPW) supplemented with 0.05% Triton X-100, which was shaken (150 rpm) at room temperature for 1 h as described in Links et al. (2014). Triton X is a surfactant that has been reported to be weakly antibacterial on its own (He et al., 2022), so it was considered safe to use for this step. The supernatant was collected and centrifuged at 4000x *g* (5,706 rpm) for 15 min. The microbial pellets were resuspended in 200 μL of TE buffer, flash-frozen in liquid nitrogen and stored at −80°C until needed. After epibiome collection, ear slices were quickly rinsed in 70% ethanol, dried on sterile Kim wipes in a sterile hood and homogenized into a fine powder using liquid nitrogen. Endobiota were collected by resuspending the powdered samples in BPW. Low centrifugation at 200x *g* (971 rpm) for 10 min allowed for separation of maize debris from the endobiota in the supernatant, which was pipetted into a new tube. Subsequent centrifugation at 4000x *g* for 15 min pelleted the endobiota and allowed them to be processed as described above for the epibiota. Microbial DNA was extracted from each of the sample types using a DNeasy Food Microbial DNA kit (Qiagen, Germantown, Maryland, United States). The DNA was then shipped to Ohio University for metagenomic sequencing and qPCR in their Genomics Facility.

### 2.4. Bacteriome (16S) and mycobiome (ITS) sequence processing

We used the 16S genomic region (V3-V4 primers) to identify taxa from the archaeal and bacterial communities (Caporaso et al., 2011; Jensen et al., 2019), and the ITS genomic region (ITS1f-ITS2 primer set) for identifying taxa from the fungal community (Smith et al., 2018). Peptide Nucleic Acid (PNA) blocks were added to the samples at the sequencing phase to lessen the impact of host genome interference as reported in Lundberg et al. (2013). To quantify the amount of K49-GFP and/or Tox4-RFP present in each tissue sample, Ohio University also conducted qPCR of our samples using primers targeting the fluorescent protein encoding genes.

To analyze the bacteriome, adapters were removed from demultiplexed reads using the Cutadapt plugin in QIIME2 v. 2021.4 (Callahan et al., 2016). Quality trimming, pairing, denoising, chimera removal, and amplicon sequence variants (ASVs) determination were conducted with the DADA2 plugin (Callahan et al., 2016) in QIIME2. For quality trimming, paired-end sequences were truncated to 271 and 220 bp for forward and reverse reads, respectively, with a median quality score of 33 and 31 for the forward and reverse reads, respectively. Taxonomic classification of the ASVs was performed by extracting the regions corresponding to the 16S 338F/806R region from the Silva 138 database (Quast et al., 2012) and training a Naive Bayes classifier using the fit-classifier-naive-bayes plugin from QIIME2. ASVs were assigned taxa using the classify-sklearn QIIME2 plugin (Pedregosa et al., 2011). ASVs identified as eukaryota, chloroplast, mitochondria, or were unassigned at the phylum level were removed from further analysis.

Only forward reads were used for the fungal ITS analysis since the reverse reads had low quality. Adapters were removed from both ends of the forward reads using the Cutadapt plugin in QIIME2 to eliminate the chance of read through since ITS regions are variable in length. The conserved region around the ITS was trimmed off using ITSxpress (Rivers et al., 2018). Removing the sequence surrounding the ITS1–ITS2 region has been shown to increase the accuracy of taxonomic classification (Nilsson et al., 2009). The reads were denoised and truncated to 160 bp using the QIIME2 DADA2 for a resulting median read quality of 38. A Naïve Bayes classifier was trained on the UNITE database v 1.12.2017 and ASVs were classified as above for bacteria. ASVs identified as chloroplast or mitochondria, or that were unassigned at the phylum level were removed from further analysis.

All combinations of maize genotype (B73, CML322), fungal strain treatment (Control, K49-GFP, Tox4-RFP, Mix) and tissue extraction (endobiota, epibiota) had at least three biological replicates except for the bacteriome of B73-Tox4-Endobiota which had only two biological replicates. Separate phylogenetic trees for bacterial and fungal ASVs were made using the fasttree QIIME2 plugin (Price et al., 2010). Rarefaction curves were made using alpha-rarefaction plugin from QIIME2 to determine a minimum sequencing depth. A cutoff of 1,500 was used for bacterial and fungal samples. This removed one bacterial sample, providing 48 bacterial samples and 50 fungal samples for further analysis. Alpha and beta diversities were calculated by rarefying datasets to the lowest total feature counts, which was 1,569. Differences between bacterial and fungal Shannon diversities were determined using a two sample Mann–Whitney U test, or a Kruskal-Wallis one-way ANOVA, followed by a pairwise Dunn test available in the R ggstatsplot package (Patil, 2021). Principle coordinate analysis (PCoA) using the Bray–Curtis dissimilarity distances was used to visualize the differences in community structure. PERMANOVA implemented in the adonis2 function from the Vegan R package (Oksanen et al., 2007) was then used to assess if the differences in the Bray-Curtis distances were significantly different between sample groups. The mantel test from the Vegan R package was used to assess correlation between the bacterial and fungal Bray-Curtis distance matrices. Differential abundance at the genus and ASV levels was assessed using ANCOM-BC (Lin and Peddada, 2020) using the formula “CornGenotype + Treatment + SampleType + CornGenotype:Treatment.” Cross-domain bacterial and fungal co-abundance networks were created using the SpiecEasi (Kurtz et al., 2015) and NetComi (Peschel et al., 2021) R packages for each maize genotype. Only ASVs with a prevalence of at least 10% were included in this analysis. The SpiecEasi package was used for estimating the associations for each network which were then passed to NetCoMi’s netConstruct function and tested for differential association using the diffnet function. Additional details of the analysis can be found at Ag Data Commons with the citation (Moore et al., 2023).

## 3. Results

### 3.1. Bacteriome diversity for maize ears of B73 and CML322

The bacteriomes (total 16S amplicons) for both inbreds included representatives from 14 of the 16 bacterial phyla, with greater representation from four groups: Actinobacteriota, Bacteroidota, Firmicutes and Proteobacteria. Overall, total relative abundance showed Proteobacteria as the predominant phylum (86.6%), followed by Firmicutes (6.5%), Actinobacteriota (3.8%) and Bacteroidota (3%). However, relative abundances of each phylum were inbred-specific. Proteobacteria was still the predominant phylum for each genotype, and it was greater in CML322 (89.9%) than in B73 (82.9%). Firmicutes were greater in relative abundance in B73 samples (11.8%) compared to those in CML322 (1.6%), but Actinobacteriota had comparable abundances in both inbreds (4.1 and 3.5%, respectively). In CML322, Bacteroidota had greater relative abundance (5%) than in B73 (1%).

Looking at the relative abundances of identified genera with a prevalence above 0.05, the bacteriomes included up to 15 that were named, while the group identified as ‘Other’ was an aggregate of genera with prevalence below 0.05 (Supplementary Figure S1). The genus *Pantoea* dominated 21 of 48 tissue samples examined with a total relative abundance of 46.1%. It dominated both maize genotypes as well, having a relative abundance of 41.9% in B73 samples and 50% in CML322 samples. Supplementary Table S1 lists the relative abundances of all bacterial genera with total relative abundances over 1%. Of the 15 genera/groups listed, 11 were Proteobacteria, three were Firmicutes and one was Bacteroidota. None of the Actinomycete bacteria made this list. Supplementary Table S2 lists 16 taxa to species level having total relative abundances over 1%. Seven of the species were Proteobacteria, four were Bacteroidota and another four were Firmicutes. Unlike the genus level, one Actinomycete bacterium (*Corynebacterium kroppenstedtii*) had a total relative abundance of 2.1%. *Pantoea ananatis* and *Burkholderia gladioli* were the most abundant species (19.3 and 15.2%, respectively). Both species had greater relative abundance values in B73 (susceptible) compared to CML322 (resistant). We estimated the impact of each treatment on bacterial alpha-diversity within each sample using Shannon’s index (Supplementary Figure S2). A Kruskal-Wallis test (Supplementary Figure S2A) showed there were significant differences in diversity among different fungal treatments (*p* = 0.006). Pairwise Dunn tests showed alpha-diversity to be greater in the control compared to Mix treatments (*p* = 0.02) and was greater in the K49-only treatment compared to the Mix treatment (*p* = 0.007; Supplementary Figure S2A). In comparing diversity between each maize genotype within each fungal treatment (Supplementary Figure S2B), CML322 showed significantly greater bacteriome diversity than B73 within the K49-only treatment (*p* = 0.005). No other bacteria alpha-diversity comparisons between maize genotypes, fungal treatments, or sample types (endobiota versus epibiota) were found to be significant. Beta-diversity (Supplementary Figure S3) of the bacteria present was assessed between samples using Bray-Curtis distance and visualized using principal coordinate analysis (PCoA). When comparing samples based on treatment, we observed a great deal of overlap, with exception of a few CML322 outliers that had been treated with K49 alone. When beta-diversity was compared between the inbreds, the samples segregated by maize genotype, despite some overlap. A permutational multivariate analysis of variance (PERMANOVA) based on Bray-Curtis distance (Table 1) showed that *A. flavus* treatment had a stronger association with bacterial variations (*R*^2^ = 0.18; *p* = 0.001) than maize genotype (*R*^2^ = 0.07; *p* = 0.001) or sample type (*R*^2^ = 0.02; *p* = 0.143). The strongest association with bacterial variation was found with the interaction of fungal treatment and inbred, having an *R*^2^ value of 0.22 (*p* = 0.001).

**Table 1:** 16S PERMANOVA results comparing differences among sample groups.

Adonis term	*R* ^2^	*p* value
Fungal treatment	0.18	0.001
Maize inbred	0.07	0.001
Sample type	0.02	0.143
Fungal treatment:Maize inbred	0.22	0.001

*R*^2^ and *p* values were calculated based on the following formula: “Bray–Curtis Dissimilarity ~ Fungal_Treatment + Corn_Genotype + Sample_Type + Fungal_Treatment:Corn_Genotype” using 999 permutations in adonis.

**Figure 1:**
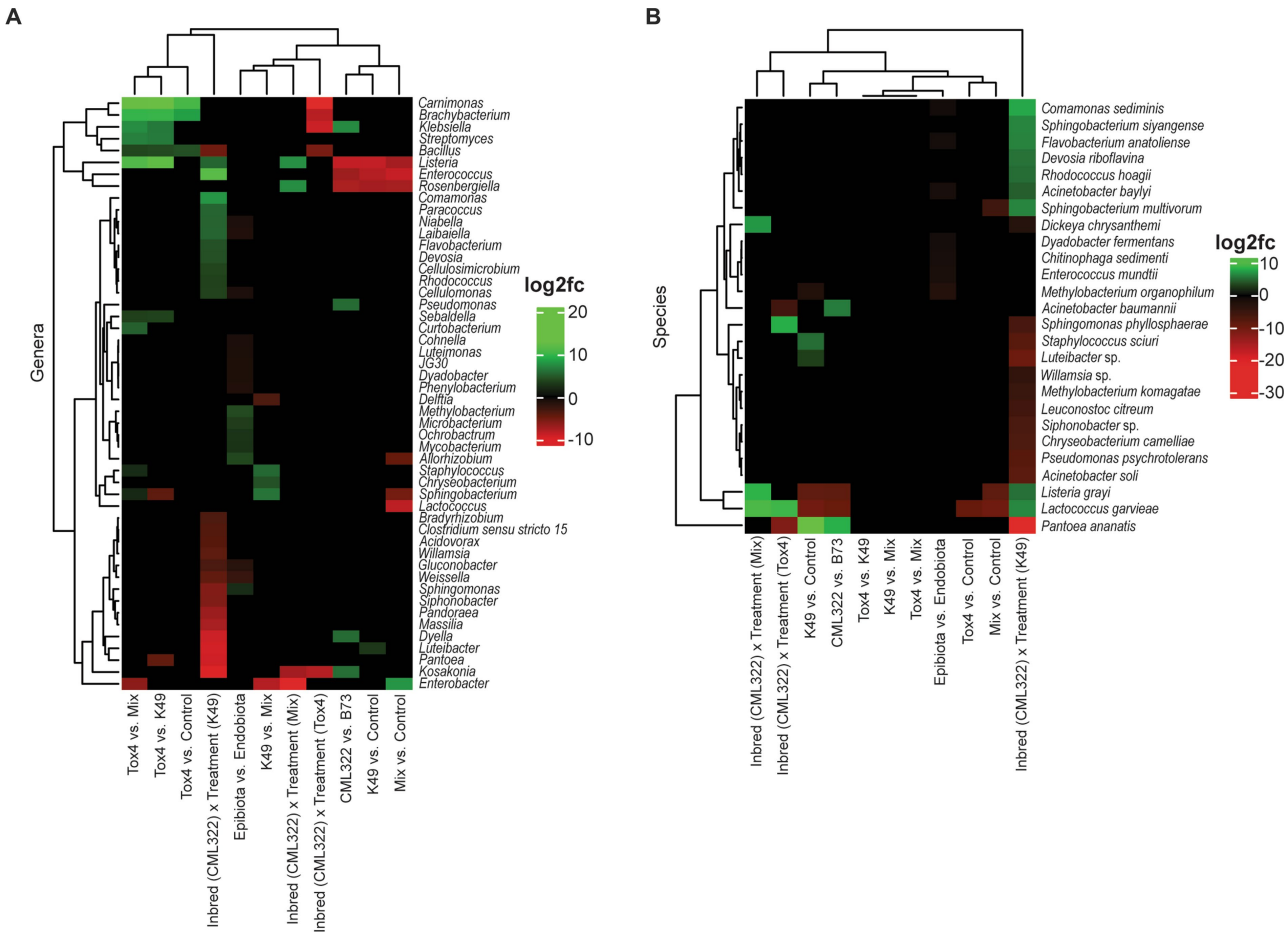
Heat map showing the log2 fold changes for 16S (bacterial) genera **(A)** and species **(B)** that were differentially abundant (at an adjusted value of *p* < 0.05) based on 11 different sets of comparisons. Positive log2 fold changes are represented in green, and negative log2 fold changes are represented in red.

**Figure 2:**
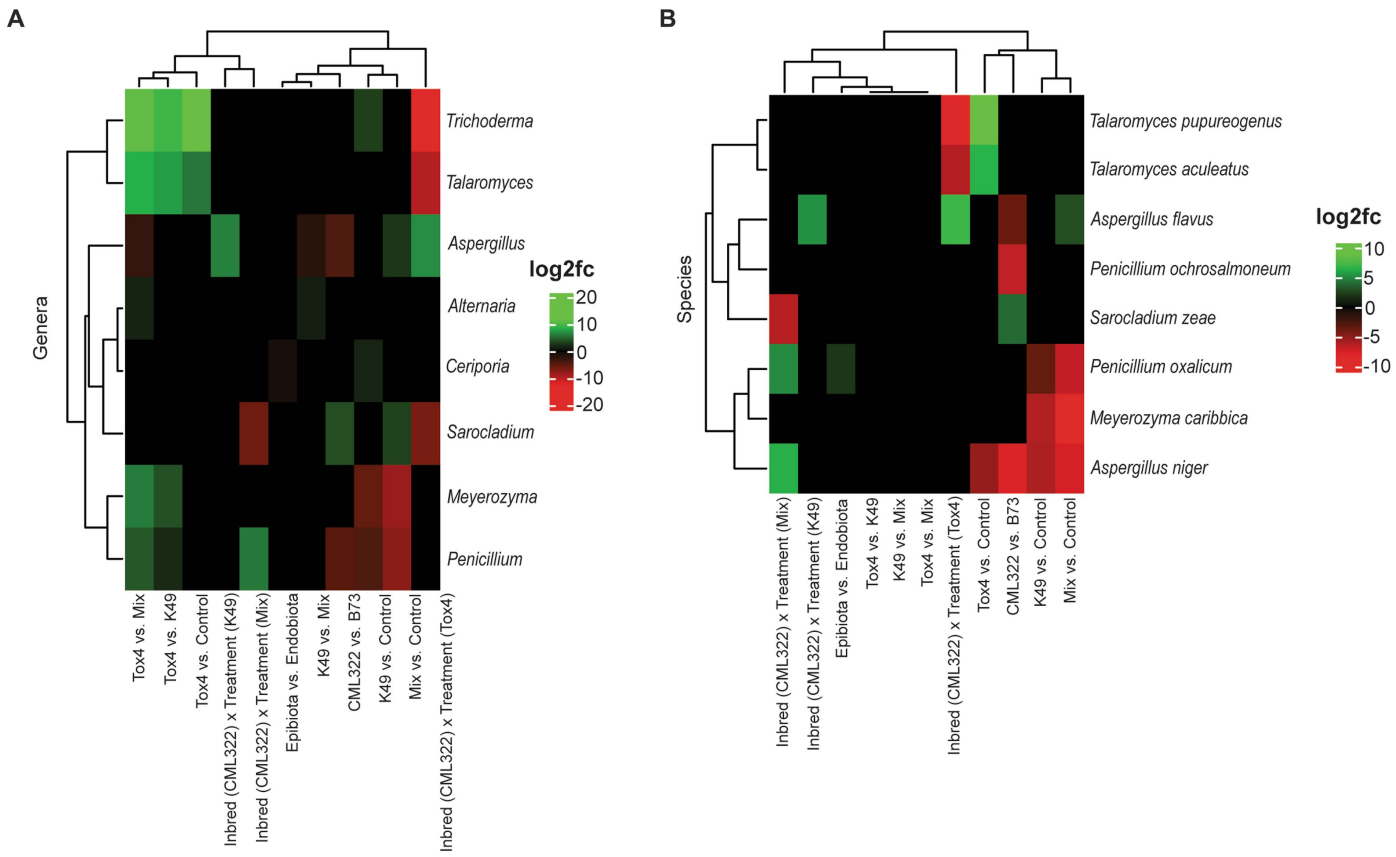
Heat map showing the log2 fold changes for ITS (fungal) genera **(A)** and species **(B)** that were differentially abundant (at an adjusted value of *p* < 0.05) based on 11 different sets of comparisons. Positive log2 fold changes are represented in green, and negative log2 fold changes are represented in red.

A differential abundance analysis was performed at the genus and species level using ANCOMBC to identify taxa that significantly differ in abundance between sample groups (Figures 1, 2). A total of 50 bacteria were differentially abundant at the genus level and 23 at the species level. Overall, the genus *Listeria* was the most dynamic, exhibiting either positive or negative differential abundance changes in seven of the 11 comparisons (Figure 1A), with the associated species, *L. grayi*, being differentially abundant in five of those seven comparisons (Figure 1B). *Lactococcus garvieae* also had seven comparisons that were differentially abundant. In comparing differential abundance between maize genotypes, the genera *Listeria*, *Rosenbergiella*, and *Enterococcus* greatly decreased with CML322 when compared to B73. Conversely, *Klebsiella*, *Pseudomonas*, *Dyella* and *Kosakonia* greatly increased with CML322 compared to B73. At the species level, *Lactococcus garvieae* showed a substantial decrease with CML322 although the genus showed no abundance differences between maize genotypes. *Klebsiella* as a genus may have experienced the greatest increase with CML322, but none of its species were found to be differentially abundant. Instead, *Pantoea ananatis* and *Acinetobacter baumannii* were the species that increased with CML322. Comparing differential abundance between each fungal treatment and the control revealed *Listeria*, *Rosenbergiella*, and *Enterococcus* having greatly decreased abundance in the Mix and K49 samples compared to the control. There were three additional genera exhibiting decreases between the Mix and control treatments (*Allorhizobium*, *Lactococcus* and *Sphingobacterium*), and only one genus increased with K49 treatment (*Luteibacter*) or with Mix treatment (*Enterobacter*). None of these genera were differentially abundant between Tox4 treated and control samples, but there was an increase in differential abundance for the genera *Carnimonas*, *Brachybacterium* and *Bacillus* with Tox4 treatment in comparison to control. Species that decreased with *A. flavus-*versus control-treated samples included *Lactococcus garvieae* (all three treatments), *Listeria grayi* (K49 and Mix treatments), *Sphingobacterium multivorum* (Mix treatment) and *Methylobacterium organophilum* (K49 treatment). Of all the *A. flavus* treatments, only K49 positively impacted differential abundance with one or more bacterial species compared to the control. Among them, *P. ananatis* had the greatest increase, followed by *Staphylococcus sciuri* and then an uncharacterized species of *Luteibacter*. Comparing changes in affected genera between Tox4 and either K49 or Mix treatments, the same seven genera were greatly increased in differential abundance with Tox4 treatment. The genus *Sphingobacterium* increased with Tox4 treatment compared to the Mix treatment but decreased with Tox4 treatment when compared to the K49 treatment. Although amplicon sequence variants (ASVs) representing species from *Listeria*, *Pseudomonas* and *Sphingobacterium* were observed having differential abundance changes, none of their relative species were shown to be differentially abundant when comparing each *A. flavus* treatment. While comparing the K49 and Mix treatments, three genera (*Staphylococcus*, *Chryseobacterium* and *Sphingobacterium*) were increased, and two genera (*Delftia* and *Enterobacter*) were decreased, with K49 treatment. Comparison of differential abundance between epibiota and endobiota showed a predominance of genera experiencing low-level change in differential abundance. Among the six genera exhibiting an increase with epibiota, compared to endobiota, *Methylobacterium* and *Mycobacterium* shared the greatest shifts in differential abundance. Eleven genera experienced decreases with epibiota versus endobiota sample types. Of those, genus *Weissella* experienced the greatest decrease. Among the species represented, none were shown to experience increases with the epibiota when compared to the endobiota. Only low-level decreases with epibiota were observed, encompassing six species. Unexpectedly, *Methylobacterium organophilum* (at the species level) showed a decrease with epibiota sample types, compared to endobiota, despite *Methylobacterium* (at the genus level) showing an increase with epibiota sample types. The last set of comparisons examined a maize-genotype-specific *A. flavus* treatment effect. The reference levels used for these comparisons were B73 for the maize genotype and the control for the fungal treatment. In this regard, the K49 treatment resulted in the greatest number of differentially abundant genera (*n* = 26), indicating that the response of these genera to fungal treatment depended on the genotype of the maize. The genus that experienced the greatest decrease in the maize-genotype-specific treatment effect was *Kosakonia*, while the genus with the greatest increase from this effect was *Enterococcus*. A similar pattern was observed at the species level, with the K49 treatment affecting differential abundance for 21 of the 23 species listed. The species having the greatest decrease from treatment effect was *Pantoea ananatis*, while *Comamonas sediminis* had the greatest increase in differential abundance. Only five genera experienced changes in differential abundance with Tox4 treatment, all of which decreased.

### 3.2. Mycobiome diversity for maize ears of B73 and CML322

Total ITS (mycobiome), for both B73 and CML322, were dominated by fungi from phylum Ascomycota (99.9%) with the remaining 0.1% comprised of Basidiomycota and Mucoromyctoa. Within each maize genotype, relative abundances of each phylum were like the overall breakdown.

The relative abundances of fungal genera with prevalence over the 0.05 cutoff were about half the number of bacterial genera, including only six genera and the ‘Other’ taxa that were below the cutoff (Supplementary Figure S4). All six genera belonged to phylum Ascomycota. Genus *Aspergillus* dominated with total relative abundance of 55.2%, and respective abundances in B73 and CML322 samples of 49.7 and 60.8% (Supplementary Table S2). This was not surprising given that 36 of the samples involved *A. flavus* treatments. Aspergilli were not as abundant in the control samples, but in B73 (21–41%) the genus was more abundant than in CML322 (<1–13%). Two genera, *Sarocladium* and *Myerozyma*, were found in high abundance in samples of both maize genotypes. Estimating the impact of each treatment on overall ITS alpha-diversity revealed no significant differences in Shannon’s diversity among the four treatment groups (Kruskal-Wallis, *p* > 0.05). However, a test performed within each maize genotype revealed statistical significance (Kruskal-Wallis, *p* = 0.04) among fungal treatments, but only within B73 (Supplementary Figure S5A). When we compared Shannon’s diversity between the two maize genotypes (Supplementary Figure S5B), there was significant difference in alpha-diversity for B73 (Mann–Whitney, *p* = 9.72e-4), whose median value was nearly double that of CML322. A comparison between control (mock) and *A. flavus* treated samples (Supplementary Figure S5C), showed significant differences in diversity between control samples (greater diversity) and the fungus treated samples (Mann–Whitney, *p* = 0.04). Comparison of diversity between maize genotypes within each of the four fungal treatments (Supplementary Figure S6A) showed significantly different mycobiome diversity, but only with the Tox4-only treatment (Mann–Whitney, *p* = 0.03). A final comparison (Supplementary Figure S6B) of fungal alpha-diversity between maize genotypes within each of the two sample types (endobiota and epibiota), showed significant differences in diversity whereby B73 diversity was greater than CML322 (Mann–Whitney, *p* ≤ 0.03). Principal coordinate analysis (PCoA) did not show a clear separation of the control and Tox4-treated samples on the first two axes, however the K49-treated samples did show a clear separation from the control and Tox4-treated samples (Supplementary Figure S7). The Mix-treated samples were distributed across the two clusters, whereby B73 samples clustered based on treatment with K49 and CML322 samples clustered based on control and Tox4 treatments. This was the only clear separation of maize genotypes within the treatment groups. PERMANOVA results (Table 2) indicated that *A. flavus* treatment had the largest effect on the ITS community composition (*R*^2^ = 0.40; *p* = 0.001), followed by the interaction between fungal treatment and maize genotype, (*R*^2^ = 0.14; *p* = 0.001). Maize genotype (*R*^2^ = 0.02; *p* = 0.090) and sample type (*R*^2^ = 0.01; *p* = 0.595) by themselves did not have significant effects on the ITS community composition.

**Table 2:** ITS PERMANOVA results comparing differences among sample groups.

Adonis term	*R* ^2^	*P* value
Fungal treatment	0.40	0.001
Maize inbred	0.02	0.090
Sample type	0.01	0.595
Fungal treatment: Maize inbred	0.14	0.001

*R*^2^ and *p* values were calculated based on the following formula: “Bray–Curtis Dissimilarity ~ Fungal_Treatment + Corn_Genotype + Sample_Type + Fungal_Treatment:Corn_Genotype” using 999 permutations in adonis.

Differential abundance analysis using ANCOMBC identified eight fungal genera (Figure 2A) and eight fungal species (Figure 2B) as significantly different between sample groups. Overall, *Aspergillus* and *Penicillium* were the most dynamic genera, each exhibiting either a positive or negative log2 fold change (log2fc) in six of the 11 comparisons. At the species level, *A. niger* was the most affected, being differentially abundant in five of the comparisons. Other *Aspergillus* and *Penicillium* species were found to be differentially abundant, but in fewer comparisons. Examining differential abundance between maize genotypes, *Aspergillus* (including both *A. flavus* and *A. niger*) and *Penicillium* (including only *P. ochrosalmoneum*) were decreased in CML322 compared to B73, while *Sarocladium* (*S. zeae*) was increased in CML322 compared to B73. Only one of the eight genera experiencing shifts in differential abundance was a basidiomycete (*Ceriporia*), which was higher in samples treated with K49 compared to the control (i.e., no K49 or Tox4 present in the treatment). It was also found to be decreased in epibiota samples compared to endobiota. A comparison of each fungal treatment to the control revealed *Trichoderma* and *Ceriporia* genera were increased in K49 treated samples, while *Meyerozyma* and *Penicillium* were decreased. Treatment with K49 resulted in decreased abundance for three species (*A. niger*, *Meyerozyma caribbica*, *Penicillium oxalicum*). *Aspergillus* and *Sarocladium* genera were increased in abundance with Mix treatment in comparison to the control, and *Meyerozyma* and *Penicillium* genera were decreased. Species associated with these two genera, as well as *A. niger*, also were decreased with Mix treatment compared to the control. However, *A. flavus* was increased with Mix treatment compared to the control. *Trichoderma* and *Talaromyces* genera positively responded to Tox4 treatment compared to the control. Although no species of *Trichoderma* were listed as having some form of change in differential abundance, two *Talaromyces* species (*T. pupureogenus* and *T. aculeatus*) were increased. *Aspergillus niger* was the only species that decreased in differential abundance with Tox4 treatment compared to the control. In comparing the *A. flavus* treatments, only two genera were not shown to exhibit some form of change in differential abundance, *Ceriporia* and *Sarocladium*. The other six genera showed a shift in differential abundance for at least one comparison. The Tox4 versus Mix comparison resulted in changes in differential abundance for six genera. Five of the six genera exhibited an increase with Tox4 treatment. Four of those five genera also responded positively to Tox4 treatment compared to the K49 treatment. When comparing the K49 and Mix treatments, *Alternaria* exhibited a low-level increase with K49 treatment and *Aspergillus* exhibited low-level decrease. Comparison of differential abundance based on sample type showed that only *Ceriporia* experienced a low-level decrease in epibiota compared to endobiota. To identify taxa influenced by the combination of maize genotype and fungal treatment, a genotype-treatment interaction term was included in the ANCOMBC formula. When we examined results from the three comparisons involving maize-genotype-specific *A. flavus* treatment effect, the Tox4 treatment resulted in the greatest number of fungal genera (4) that showed a change in differential abundance. Genus *Aspergillus* exhibited the only increase from the maize-genotype-specific treatment effect, while *Trichoderma* had the greatest decrease. At the species level, only *A. flavus* had a positive shift in differential abundance, while two *Talaromyces* species experienced negative shifts from Tox4 treatment. *Aspergillus* was the only genus to increase with maize-genotype-specific treatment effect involving K49 and experienced no shift in differential abundance with the maize-genotype-specific effect involving Mix treatment. Similarly, *A. flavus* was the only species to increase in abundance with K49 treatment, while it neither increased nor decreased with Mix treatment. Only *Sarocladium* (decrease) and *Penicillium* (increase) were affected by Mix treatment in this comparison involving maize-genotype-specific *A. flavus* treatment effect. Species from each genus exhibited similar directional shifts in differential abundance. Although the genus *Aspergillus* showed no shifts in differential abundance, *A. niger* increased with Mix treatment.

### 3.3. Abundance and impact of *Aspergillus flavus* in maize samples

**Figure 3:**
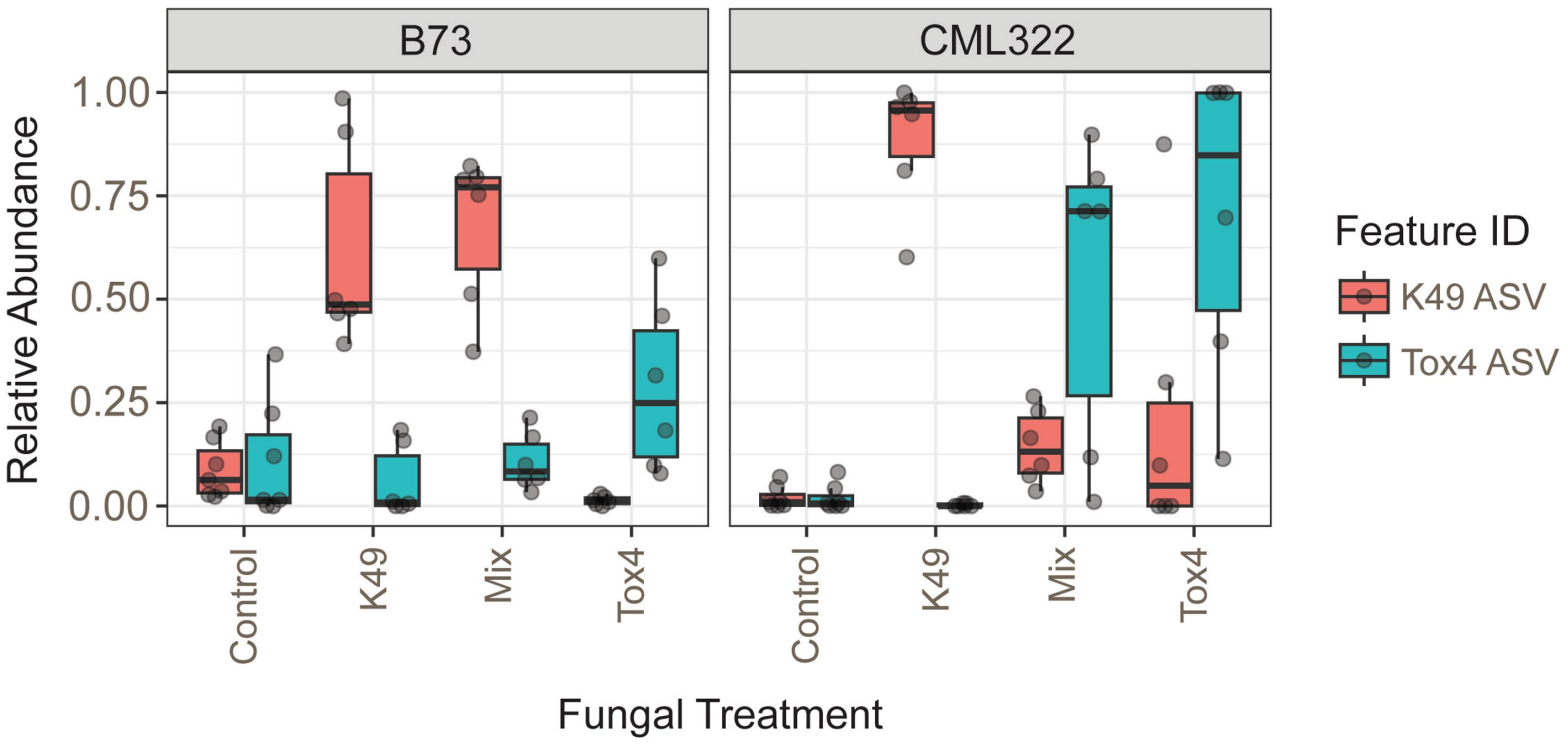
Box plots showing the relative abundances of the 2 *A. flavus* ASVs (K49 and/or Tox4) present in each of the four treatments used for both maize inbreds (B73 and CML322).

There were six different *A. flavus* amplicon sequence variants (ASVs) identified with a prevalence over 5%, but only two of them had a relative abundance greater than 1% in any sample. The two predominant ASVs differed by one nucleotide and allowed us to distinguish between the two fungal strains used in the treatments. The 06ee4aa94b2b7521c7b13c1fb07879bf and f238a589635f01ddc3a300a2f6e63322 ASVs identically matched the K49 genome and Tox4 genome, respectively. These 2 ASVs made up a substantial portion of the ITS counts. Each respective ASV in K49 and Tox4 treatments had a median relative abundance of 85 and 43%, respectively. K49 and Tox4 ASVs were present in low levels in both control samples, but both were present at greater relative abundance in B73 (Figure 3). The K49-only treatment in B73 showed the biocontrol strain’s ASV had much greater relative abundance than Tox4 (as expected), but also showed the presence of some samples with the Tox4 ASV at low levels as well. In CML322, the Tox4 ASV was absent in samples that were treated only with K49. B73 samples treated with Tox4 alone showed no presence of K49; however, the K49 ASV was present in low levels in the CML322 samples that were supposed to contain only the Tox4 ASV. The differential abundance results at the ASV level revealed a significant genotype-treatment interaction effect for the K49 ASV in the K49 treatment of CML322. When we tested whether the relative abundances of the two *A. flavus* ASVs were different in the Mix-treated samples (using a non-parametric Mann–Whitney U test), we found they were not significantly different (Supplementary Figure S8A). However, when we tested whether the relative abundances of the two *A. flavus* ASVs were different within the two maize genotypes, we found the K49 ASV was present at significantly greater counts than the Tox4 ASV in B73 (*p* = 5.07e-3; Supplementary Figure S8B). However, the abundance of Tox4 was greater in the Mix treatment of CML322.

Looking at cross domain network associations for each introduced *A. flavus* strain at the ASV level revealed no coabundance associations with other fungi. K49 had a moderately positive association with bacterial genus *Pantoea* (no species identified) in B73, while this same *A. flavus* strain had a negative association with *Lysteria grayi* (Table 3). Tox4 had a stronger negative association with bacteria from order Lactobacillales in B73 (no genus or species was identified). For CML322, only Tox4 had associations with bacteria. Tox4 had a strong positive association with *Staphylococcus sciuri* and a weakly positive association with a representative of *Cutibacterium* (species not identified). It also had weakly negative associations with representatives of *Klebsiella* and *Microbacterium* (species not identified).

**Table 3:** Cross domain network associations based on maize inbred with edge weights between each introduced *A. flavus* strain and its associated taxa.

Maize inbred	Flavus ASV	Edge weight	Order	Genus	Species
B73	K49	0.04270460	Enterobacterales	*Pantoea*	*NA*
B73	K49	−0.03066922	Lactobacillales	*Listeria*	*Listeria grayi*
B73	Tox4	−0.07616467	Lactobacillales	*NA*	*NA*
CML322	Tox4	0.07026076	Staphylococcales	*Staphylococcus*	*Staphylococcus sciuri*
CML322	Tox4	0.02293734	Propionibacteriales	*Cutibacterium*	*NA*
CML322	Tox4	−0.01190633	Enterobacterales	*Klebsiella*	*NA*
CML322	Tox4	−0.01617679	Micrococcales	*Microbacterium*	*NA*

## 4. Discussion

### 4.1. Characterizing the dominant bacteria on maize seed

Only 15 bacterial genera were found to have total relative abundances above 1%. Some have known pathogenic capabilities, while others are considered beneficial to maize. Genus *Pantoea* was the dominant taxon among the 16S sequences (Supplementary Table S1). Within the two maize inbreds, this genus represented 41.9% of bacterial taxa in B73 and 50% in CML322. The only species of *Pantoea* with a high abundance was *P. ananatis*, having the highest total relative abundance (19.3%) of all bacteria present, and was the most abundant within each maize genotype (Supplementary Table S2). This species has been reported to negatively impact maize growth by causing diseases such as leaf spot (Paccola-Meirelles et al., 2001), brown stalk rot (Goszczynska et al., 2007) and leaf streak (Zhang et al., 2023). Its abundance was greatly impacted by K49 treatment in a maize genotype-dependent manner. *Pantoea ananatis* decreased from K49 treatment relative to the control in CML322 but increased from this same treatment in B73. Based on relative abundance in each inbred, the genus with the second greatest abundance in B73 was *Enterobacter* (10.1%). This genus also experienced significant changes in differential abundance for some comparisons, mostly those involving the Mix treatment. Many *Enterobacter* species are considered human pathogens of clinical importance, partially due to their antibiotic resistance (Davin-Regli et al., 2019), but some are beneficial. For example, *E. cloacae* has been reported as an endophyte of maize with proven antifungal properties against mycotoxin producing fungi such as *Fusarium* and *Aspergillus* (Hinton and Bacon, 1995), and volatiles from another species, *E. asburiae*, have been shown to reduce growth and aflatoxin production by *A. flavus* (Gong et al., 2019). However, the *Enterobacter* taxa in our dataset were unable to be identified at the species level. Although *Burkholderia* as a genus only contributed 3% to total relative abundance, one of its species (*B. gladioli*) had the second highest overall abundance with 15.2%. This species also had the second greatest relative abundance in B73 (19.5%), more than double its abundance in CML322. Neither level (genus nor species) for this taxon was shown to experience a change in differential abundance across our 11 comparisons. Despite being a root endophyte with plant protection and growth promotion effects in maize (Gunjal and Kapadnis, 2013; Shehata and Raizada, 2017; Dos Santos et al., 2022), *B. gladioli* also has pathovars (pathogenic variants) implicated in causing maize ear rot (Lu et al., 2007) and other plant diseases (Moon et al., 2017). In CML322, the genus with the second greatest abundance was *Klebsiella* (15.9%). Species from this genus (e.g., *K. pneumoniae*) also can cause animal and human diseases and have resistance to antibiotics (Zadoks et al., 2011; Ballén et al., 2021). Although not beneficial to humans, *K. pneumoniae* is reported to offer endophytic benefits to maize growth (Chelius and Triplett, 2000). Additional species have been shown to be beneficial to maize as an endophyte. For example, both *K. jilinsis* and *K. variicola* promote growth in maize and help to prevent disease (Yang and Yang, 2020; Zhang et al., 2021). As a genus, *Klebsiella* experienced differential abundance changes in several of our comparisons. However, no species of *Klebsiella* experienced notable changes in differential abundance, so it is unclear if any of its species were positively impacted by the introduction of the K49 biocontrol strain. Although there are no known reports of *Klebsiella* species having antifungal properties against *A. flavus*, the fact that the relative abundance of this genus in CML322 was 13.5% greater than in B73 may contribute to its resistant phenotype. Further research would be required to confirm or refute this possibility. One other species with greater than 10% relative abundance in B73 was *Listeria grayi* (11.9%), a much greater abundance than found in CML322. Conversely, *Sphingopbacterium siyangense* had relative abundance of 12%, the second highest in CML322, with much greater abundance than it had in B73. At both the genus and species levels, these two taxa experienced significant changes in their respective differential abundances in at least one of the 11 comparisons. Supplementary Table S3 lists some of the bacterial genera mentioned here along with additional information about them and associated references.

There were nine species (including *L. grayi* and *S. siyangense*) that showed an increase in abundance with K49 treatment in CML322 compared to B73 (Figure 1B), including representatives from all four of the predominant bacterial phyla. A literature search showed none of these species having a documented association with maize plants or *A. flavus*. There are closely related species to those listed in Figure 2 (e.g., from genus *Sphingobacterium*) that offer growth benefits to maize (Mehnaz et al., 2007; Kämpfer et al., 2016). However, these beneficial species (*S. canadense* and *S. zeae*) were either absent in our dataset or not identifiable at the species level. In addition to *P. ananatis*, there were 11 species that showed a decrease in abundance with K49 treatment in CML322 compared to B73, also having representatives from the four dominant phyla. One of those species was *Dickeya chrysanethemi*. A pathovar of this species (pv. *zeae*) was renamed as *D. zeae* (Samson et al., 2005) and it has been associated with maize stalk rot for nearly seven decades (Sabet, 1954; Caplik et al., 2022). It is unclear if the *D. chrysanthemi* listed in Figure 2 is identical to *D. zeae*. However, it is clear this potentially harmful bacterium had a decrease in abundance in CML322, compared to B73, when treated with K49. Additionally, three species reported to benefit maize plants also experienced a decrease in abundance (albeit at a low-level) in CML322 compared to B73 when treated with K49. These beneficial organisms were *Chryseobacterium camelliae* (Wu et al., 2021), *Pseudomonas psychrotolerans* (Kubi et al., 2021), and *Siphonobacter* sp. (Wu et al., 2021). If these three species are important contributors to the resistant phenotype of CML322, their decreased abundance in response to the biocontrol strain might lower the resistance of this maize genotype to other pathogenic fungi. However, their increased abundances in B73 suggest the introduction of the biocontrol strain is a positive outcome for this *A. flavus* susceptible genotype. The other eight species exhibiting a decrease in differential abundance had no documented association with maize or *A. flavus*.

Abundances of potentially harmful genera such as *Pantoea* (*P. ananatis*) and *Dickeya* (*D. chrysanthemi*) showed a maize genotype dependent decrease in CML322 with K49 biocontrol treatment. This suggests there is added benefit to growers from planting an *A. flavus* resistant genotype and applying the K49 biocontrol strain. However, there were also some beneficial bacteria that decreased in abundance by the same treatment. This suggests there still are no known bacteria that work well with *A. flavus* biocontrol strains and can be used as helper organisms to enhance biocontrol efficacy. Further research is needed to identify such bacterial species that respond well to the biocontrol strain and confirm their potential benefit to maize.

### 4.2. Characterizing the dominant fungi on maize seed

Five fungal genera (all Ascomycota) had total relative abundances at 1% or more, of which *Aspergillus* (55.2%) dominated the ITS sequences for treated samples (Supplementary Table S4; Supplementary Figure S4). The dominant species was *A. flavus*, having a total relative abundance of 56.7% (Supplementary Table S5). Broken down by maize genotype it had an abundance of 52.5% in B73 and an abundance of 60.8% in CML322. These findings were expected given that we inoculated our maize ears with *A. flavus* strains. Even without manual introduction, this genus is ubiquitous and certain species are commonly associated with maize, such as *A. flavus* and *A. niger* (Goko et al., 2021). In fact, *A. niger* was one of the other species with total relative abundance over 1% and was increased in B73 compared to CML322. The genus with the second greatest total relative abundance was *Sarocladium* (25.2%), and its associated species *S. zeae* had a similar value for total relative abundance. At both genus and species levels, this taxon was present at greater abundance in CML322 compared to B73. *Sarocladium zeae* was the only species from this genus exhibiting differential abundance changes, which increased in CML322 compared to B73 (Figure 2B). A known endophyte of maize, *S. zeae* (formerly *Acremonium zeae*) produces inhibitory compounds that reduce prevalence and mycotoxin production by fungal pathogens such as *A. flavus* and *Fusarium verticillioides* (Wicklow et al., 2005; Gao et al., 2020). Perhaps the increased abundance of *Sarocladium*, particularly *S. zeae*, in CML322 contributes to its innate ability to resist infection and subsequent aflatoxin contamination by *A. flavus*. Further investigation is needed to confirm or refute this. There was no change in differential abundance of *S. zeae* from treatment with K49, suggesting that use of *A. flavus* biocontrol strains like K49 will not inhibit the abundance of this beneficial fungus. The other three genera with total relative abundances above 1% (*Meyerozyma*, *Talaromyces* and *Trichoderma*) were more abundant in B73 than in CML322 although these values for genus *Meyerozyma* were not that different. Genus *Meyerozyma* had the greatest relative abundance values in our control samples, even greater than genus *Aspergillus*. The species *Meyerozyma caribbica* had the third highest abundance values (13.5%). Differential abundance analyzes revealed an increase in genus *Meyerozyma* with Tox4 compared to the other *A. flavus* treatments (K49 and Mix). However, at the species level there were no observed increases in differential abundance of *M. caribbica* associated with Tox4. *Meyerozyma caribbica* (synonym *Pichia caribbica*) is a yeast fungus with inhibitory properties against phytopathogenic ascomycetes such as *Penicillium expansum* (Qiu et al., 2022) and *Colletotrichum gloeosporioides* (González-Gutiérrez et al., 2021), and has been shown to offer growth-promoting benefits to maize plants (Jan et al., 2019). A literature search revealed no reports involving interactions between *M. caribbica* (or *P. caribbica*) and *A. flavus*, although a different species of *Pichia* (*P. anomala*) was shown to produce at least one volatile compound that is inhibitory to *A. flavus* and aflatoxin production (Hua et al., 2014). Our analysis revealed that *M. caribbica* decreased in differential abundance from the K49 and Mix treatments compared to the control (at both genus and species levels), suggesting that application of K49 might be antagonistic to this taxon at some level. Having relatively low abundance levels (albeit above 1%), the genera *Trichoderma* and *Talaromyces* increased in differential abundance for all three treatments involving Tox4 (in comparison to K49, Mix and Control treatments). There was also a maize genotype specific Tox4 treatment effect where both genera showed a significant decrease in abundance from the Tox4 treatment in CML322 compared to this same treatment in B73. Given the treatment effect decreases in *Trichoderma* and *Talaromyces* were only found with Tox4 in CML322 samples, it is possible their decreases resulted from CML322’s resistant phenotype (i.e., broadly antifungal beyond *A. flavus*). However, many species of *Trichoderma* have been tested as biocontrol agents to inhibit *A. flavus* growth and reduce aflatoxin levels (Ren et al., 2022). Although no species of *Trichoderma* had a relative abundance above 1% or a positive change in differential abundance associated with Tox4, the genus’ increase in differential abundance with B73 could indicate a response to the presence of this highly aflatoxigenic strain. Additionally, a low-level increase in differential abundance of *Trichoderma* from the K49 treatment compared to the control showed that biocontrol application did not hinder the proliferation of potentially beneficial *Trichoderma*. Two *Talaromyces* species (*T. aculeatus* and *T. purpureogenus*) were listed as experiencing differential abundance changes in response to treatment with Tox4 (increases), but only compared to the control, while also experiencing similar decreases in differential abundance from Tox4 treatment in CML322 compared to the same treatment in B73. *Talaromyces aculeatus* produces derivatives of azaphilones (Ren et al., 2017), which are metabolites reported to have antimicrobial properties (Osmanova et al., 2010), and *T. purpureogenus* has potential as a biocontrol agent against *Fusarium oxysporum* (Tian et al., 2020). A literature search involving either species resulted in no identified direct associations with either maize or *A. flavus*, so labeling them as helper organisms to *A. flavus* biocontrol requires further investigation.

*Penicillium ochrosalmoneum*, experienced a sizeable decrease in abundance in CML322 compared to B73. This fungus has a known association with maize and has the potential to contaminate kernels with a mycotoxin known as citreoviridin (Wicklow et al., 1988), and its teleomorph (*Eupenicillium ochrosalmoneum*) has been reported to parasitize conidial heads of closely related species from section *Flavi*, including *A. flavus*, with a high level of host specificity (Horn and Peterson, 2008). Decreased presence of *P. ochrosalmoneum* (and *A. niger*) associated with CML322 supports the potential for this maize genotype to be resistant to mycotoxin producing fungi beyond *A. flavus*. The other *Penicillium* species (*P. oxalicum*) experienced differential abundance changes in four of the 11 comparisons, including decreases from both K49 and Mix treatments (compared to the control), a low-level increase in epibiota compared to endobiota, and a moderate increase in CML322 from the Mix treatment compared to this same treatment in B73. Although not considered mycotoxigenic, this species is a known pathogen of maize that causes seedling stunting and blight (Halfon-Meiri and Solel, 1990). Supporting our observed increase in differential abundance in epibiota compared to endobiota, Halfon-Meiri and Solel (1990) reported that *P. oxalicum* was only found on the surfaces of examined maize kernels.

The relative abundances of two fungal species that are known to offer benefits to maize (*S. zeae* and *M. caribbica*) remained relatively high despite the introduction of the *A. flavus* strains, especially Tox4. Additionally, *Meyerozyma* fungi may not have the fitness traits to compete against an aggressive fungus such as *A. flavus* since its presence was greatly diminished in treated samples for both maize genotypes (compared to the controls). However, further research is necessary to confirm or refute these possibilities. Regardless of their associations with one another, their beneficial traits and retained presence in maize suggest they might be potential helpers to *A. flavus* biocontrol and the maize plant. Further research from this lab will examine microbiomes of other maize niches, such as the rhizosphere, upon introduction of *A. flavus* biocontrol. Additionally, there will be testing of specific bacteria and fungi from these microbiomes for their abilities to improve the aflatoxin-reductive potential of biocontrol strains.

## 5. Conclusion

Despite causing significant changes in the overall microbial community, the microbiomes associated with kernels of maize plants that were treated with *A. flavus* strains, especially the K49 biocontrol, in many cases harbored bacterial and fungal species that hold promise as biocontrol enhancer organisms. These microbes on their own may not be effective against aflatoxigenic fungi, but in concert with *A. flavus* biocontrol strains they may further enhance its efficacy.

## Data availability statement

The raw reads have been submitted to the ENA and are available under project PRJEB66233.

## Author contributions

GM: Conceptualization, Methodology, Project administration, Writing – original draft, Writing – review & editing. SC: Investigation, Methodology, Writing – review & editing. BM: Data curation, Formal analysis, Writing – review & editing. MG: Conceptualization, Writing – review & editing. JC: Conceptualization, Writing – review & editing. KR: Conceptualization, Writing – review & editing.
