# Supplementary material for: Microbiota of maize kernels as influenced by Aspergillus flavus infection in susceptible and resistant inbreds
Source: Front Microbiol. 2023 Nov 6;14:1291284. doi: 10.3389/fmicb.2023.1291284 (PMC10657875; doi:10.3389/fmicb.2023.1291284)
Supplement: Supplementary file 1 [file Table_1.DOCX]

**Table 1S.** 16S genera with total relative abundance above 1% with breakdown by maize inbred

| **Genus ^a^** | **Total Relative Abundance** | **Relative abundance B73** | **Relative abundance CML322** |
| --- | --- | --- | --- |
| *Pantoea* (P) | 0.461 | 0.419 | 0.500 |
| *Klebsiella* (P) | 0.094 | 0.024 | 0.159 |
| *Enterobacter* (P) | 0.051 | 0.101 | 0.005 |
| *Carnimonas* (P) | 0.041 | 0.085 | 0.000 |
| *Burkholderia-Caballeronia-Paraburkholderia* (P) | 0.030 | 0.048 | 0.013 |
| *Serratia* (P) | 0.029 | 0.033 | 0.026 |
| *Stenotrophomonas* (P) | 0.029 | 0.014 | 0.043 |
| *Lactococcus* (F) | 0.024 | 0.048 | 0.003 |
| *Sphingobacterium* (B) | 0.024 | 0.007 | 0.039 |
| *Achromobacter* (P) | 0.023 | 0.008 | 0.036 |
| *Rosenbergiella* (P) | 0.022 | 0.047 | 0.000 |
| *Listeria* (F) | 0.019 | 0.040 | 0.000 |
| *Allorhizobium-Neorhizobium-Pararhizobium-Rhizobium* (P) | 0.017 | 0.009 | 0.023 |
| *Ochrobactrum* (P) | 0.017 | 0.013 | 0.020 |
| *Enterococcus* (F) | 0.010 | 0.013 | 0.007 |

^a^ Phylum to which each genus belongs: B (Bacteroidota), F (Firmicutes), P (Proteobacteria/Pseudomonadota)
