# Supplementary material for: Microbiota of maize kernels as influenced by Aspergillus flavus infection in susceptible and resistant inbreds
Source: Front Microbiol. 2023 Nov 6;14:1291284. doi: 10.3389/fmicb.2023.1291284 (PMC10657875; doi:10.3389/fmicb.2023.1291284)
Supplement: Supplementary file 2 [file Table_2.DOCX]

**Table 2S.** 16S species with total relative abundance above 1% with breakdown by maize inbred

| **Species ^a^** | **Total Relative Abundance** | **Relative abundance B73** | **Relative abundance CML322** |
| --- | --- | --- | --- |
| *Pantoea ananatis* (P) | 0.193 | 0.207 | 0.173 |
| *Burkholderia gladioli* (P) | 0.152 | 0.195 | 0.091 |
| *Listeria grayi* (F) | 0.070 | 0.119 | 0.000 |
| *Lactococcus lactis* (F) | 0.068 | 0.077 | 0.056 |
| *Sphingobacterium siyangense* (B) | 0.050 | 0.001 | 0.120 |
| *Lactococcus garvieae* (F) | 0.043 | 0.073 | 0.000 |
| *Sphingobacterium thalpophilum* (B) | 0.032 | 0.000 | 0.079 |
| *Sphingomonas phyllosphaerae* (P) | 0.030 | 0.000 | 0.073 |
| *Acinetobacter baumannii* (P) | 0.029 | 0.000 | 0.070 |
| *Pseudomonas psychrotolerans* (P) | 0.026 | 0.008 | 0.052 |
| *Comamonas sediminis* (P) | 0.023 | 0.000 | 0.056 |
| *Corynebacterium kroppenstedtii* (A) | 0.021 | 0.036 | 0.000 |
| *Flavobacterium anatoliense* (B) | 0.019 | 0.000 | 0.047 |
| *Sphingobacterium multivorum* (B) | 0.015 | 0.012 | 0.019 |
| *Staphylococcus sciuri* (F) | 0.015 | 0.026 | 0.000 |
| *Devosia riboflavina* (P) | 0.014 | 0.000 | 0.035 |
| uncultured *Tistrella* (P) | 0.013 | 0.023 | 0.000 |
| uncultured bacterium | 0.010 | 0.014 | 0.003 |

^a^ Phylum to which each species belongs: A (Actinobacteria), B (Bacteroidota), F (Firmicutes), P (Proteobacteria/Pseudomonadota)
