# Supplementary material for: Microbiota of maize kernels as influenced by Aspergillus flavus infection in susceptible and resistant inbreds
Source: Front Microbiol. 2023 Nov 6;14:1291284. doi: 10.3389/fmicb.2023.1291284 (PMC10657875; doi:10.3389/fmicb.2023.1291284)
Supplement: Supplementary file 4 [file Table_4.docx]

**Table 4S.** ITS genera with total relative abundance above 1% with breakdown by maize inbred

| **Genus ^a^** | **Total Relative Abundance** | **Relative abundance B73** | **Relative abundance CML322** |
| --- | --- | --- | --- |
| *Aspergillus* (A) | 0.552 | 0.497 | 0.608 |
| *Sarocladium* (A) | 0.252 | 0.226 | 0.279 |
| *Meyerozyma* (A) | 0.124 | 0.148 | 0.101 |
| *Talaromyces* (A) | 0.040 | 0.076 | 0.003 |
| *Trichoderma* (A) | 0.013 | 0.026 | 0.001 |

^a^ Phylum to which each genus belongs: A (Ascomycota)
