# Supplementary material for: Microbiota of maize kernels as influenced by Aspergillus flavus infection in susceptible and resistant inbreds
Source: Front Microbiol. 2023 Nov 6;14:1291284. doi: 10.3389/fmicb.2023.1291284 (PMC10657875; doi:10.3389/fmicb.2023.1291284)
Supplement: Supplementary file 5 [file Table_5.docx]

**Table 5S.** ITS species with total relative abundance above 1% with breakdown by maize inbred

| **Species ^a^** | **Total Relative Abundance** | **Relative abundance B73** | **Relative abundance CML322** |
| --- | --- | --- | --- |
| *Aspergillus flavus* (A) | 0.567 | 0.525 | 0.608 |
| *Sarocladium zeae* (A) | 0.259 | 0.234 | 0.284 |
| *Meyerozyma caribbica* (A) | 0.135 | 0.168 | 0.102 |
| *Aspergillus niger* (A) | 0.011 | 0.021 | 0.000 |
| *Talaromyces purpureogenus* (A) | 0.010 | 0.019 | 0.000 |

^a^ Phylum to which each genus belongs: A (Ascomycota)
