# Supplementary figures and images for: Microbiota of maize kernels as influenced by Aspergillus flavus infection in susceptible and resistant inbreds
Source: Front Microbiol. 2023 Nov 6;14:1291284. doi: 10.3389/fmicb.2023.1291284 (PMC10657875; doi:10.3389/fmicb.2023.1291284)

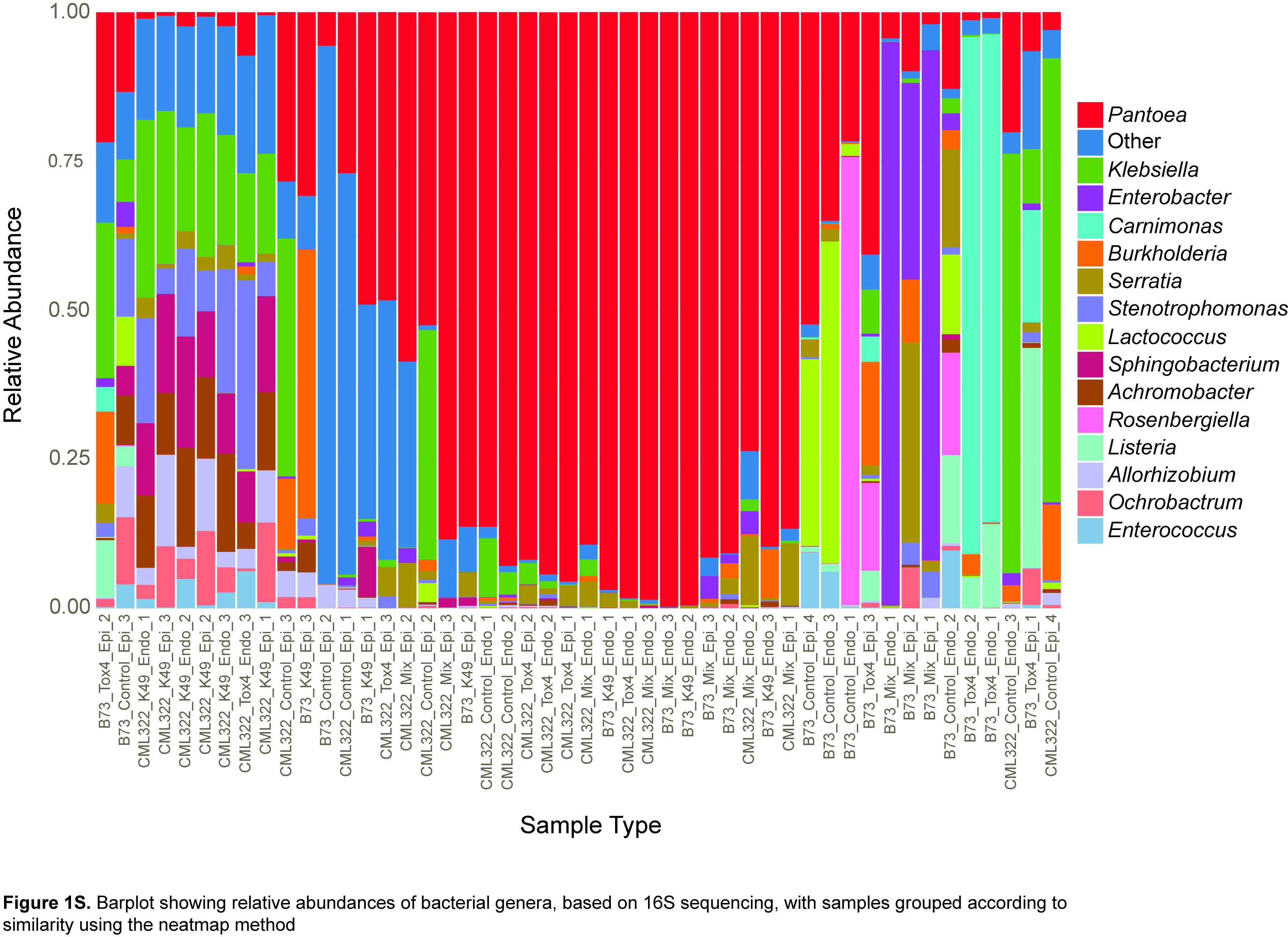

Supplement: Supplementary file 6 [file Image_1.JPEG]

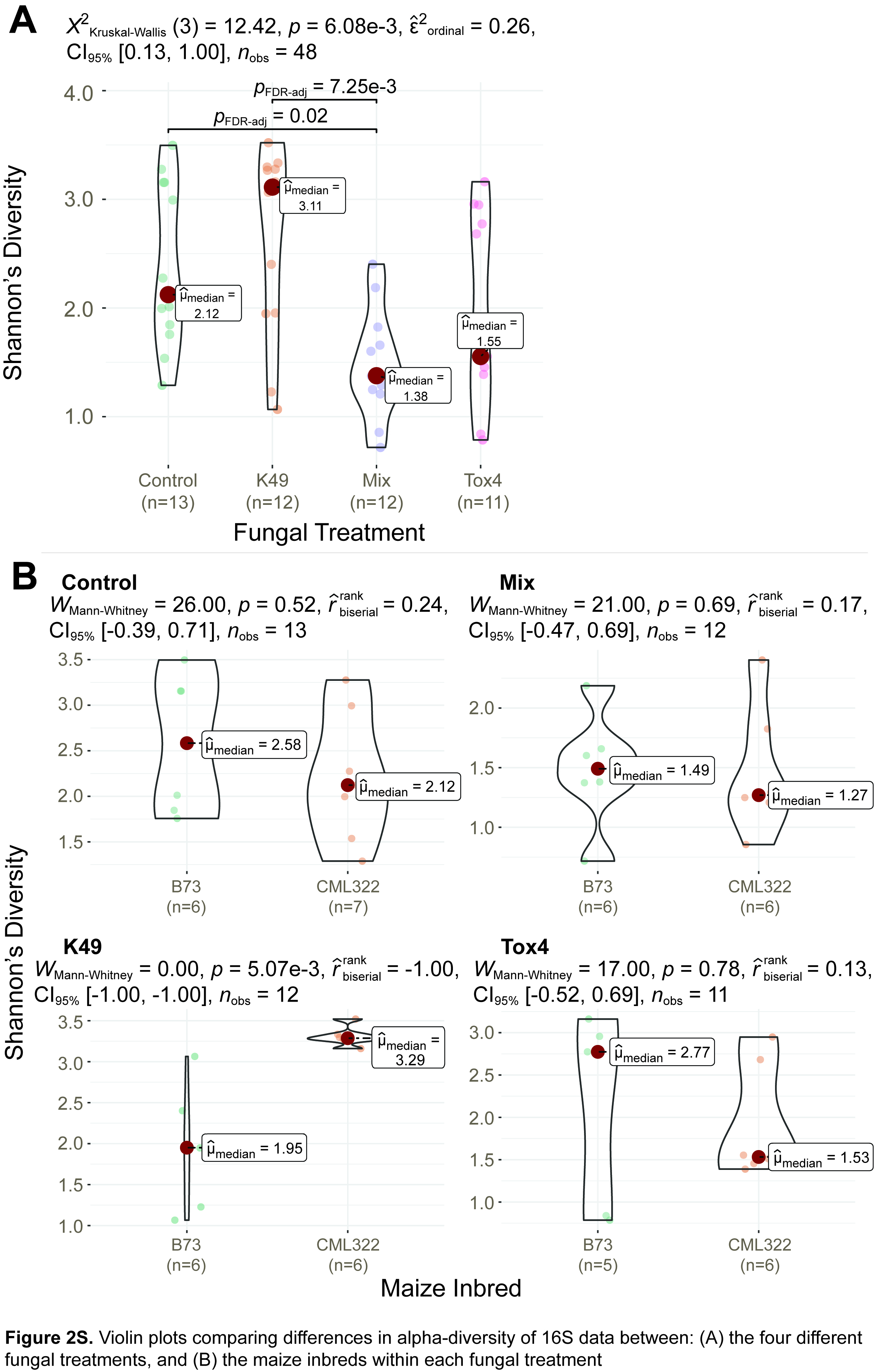

Supplement: Supplementary file 7 [file Image_2.JPEG]

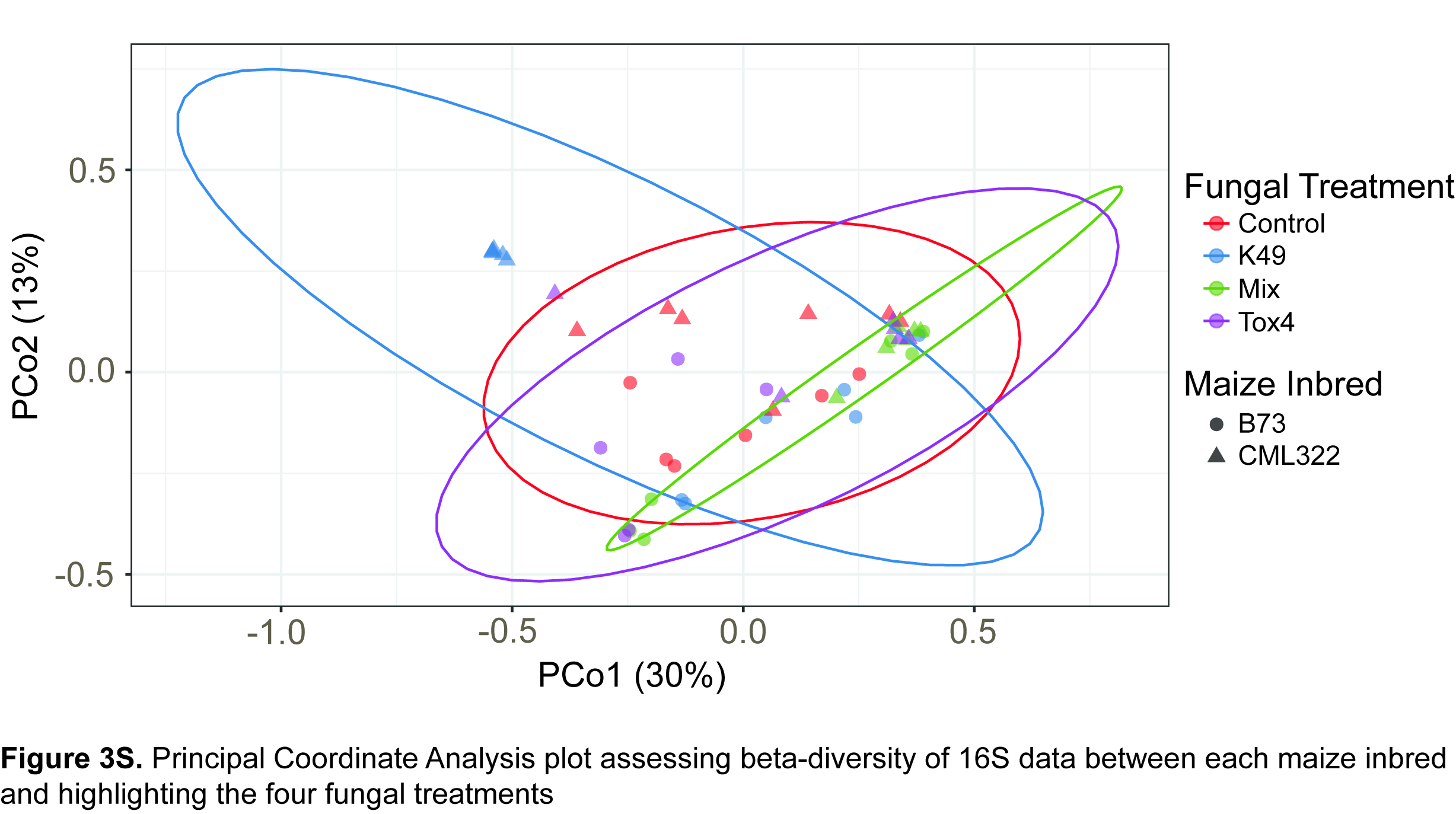

Supplement: Supplementary file 8 [file Image_3.JPEG]

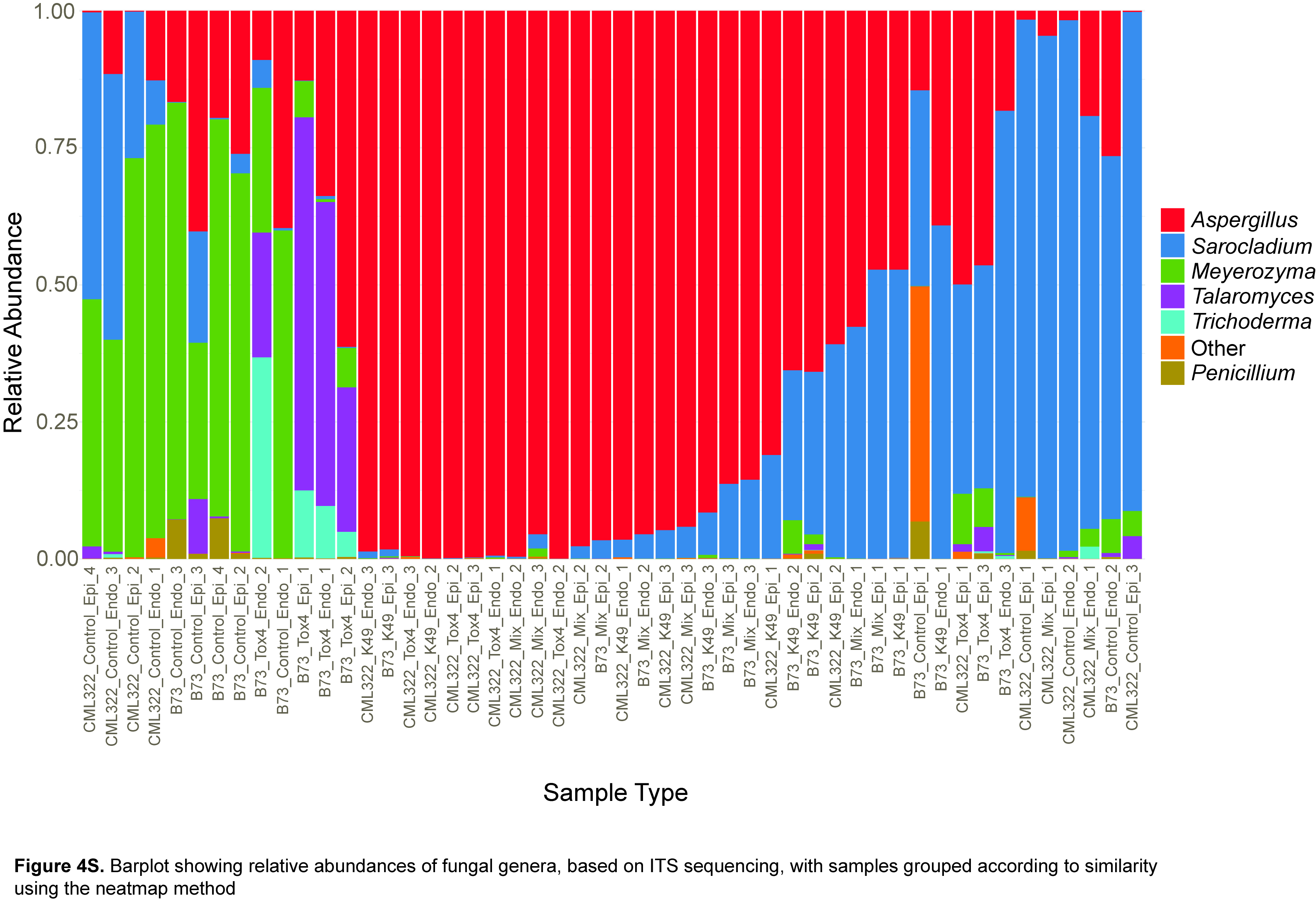

Supplement: Supplementary file 9 [file Image_4.JPEG]

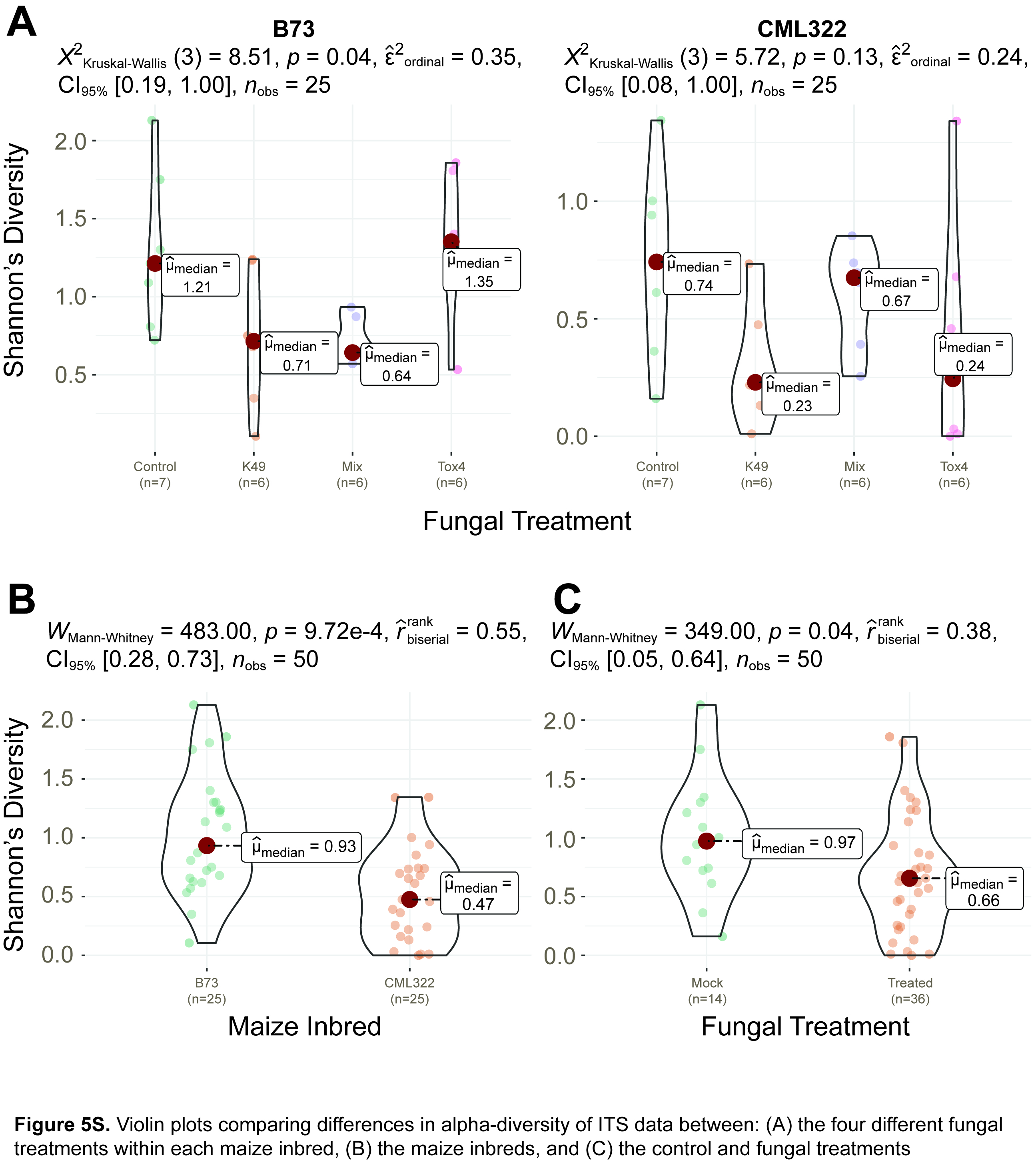

Supplement: Supplementary file 10 [file Image_5.JPEG]

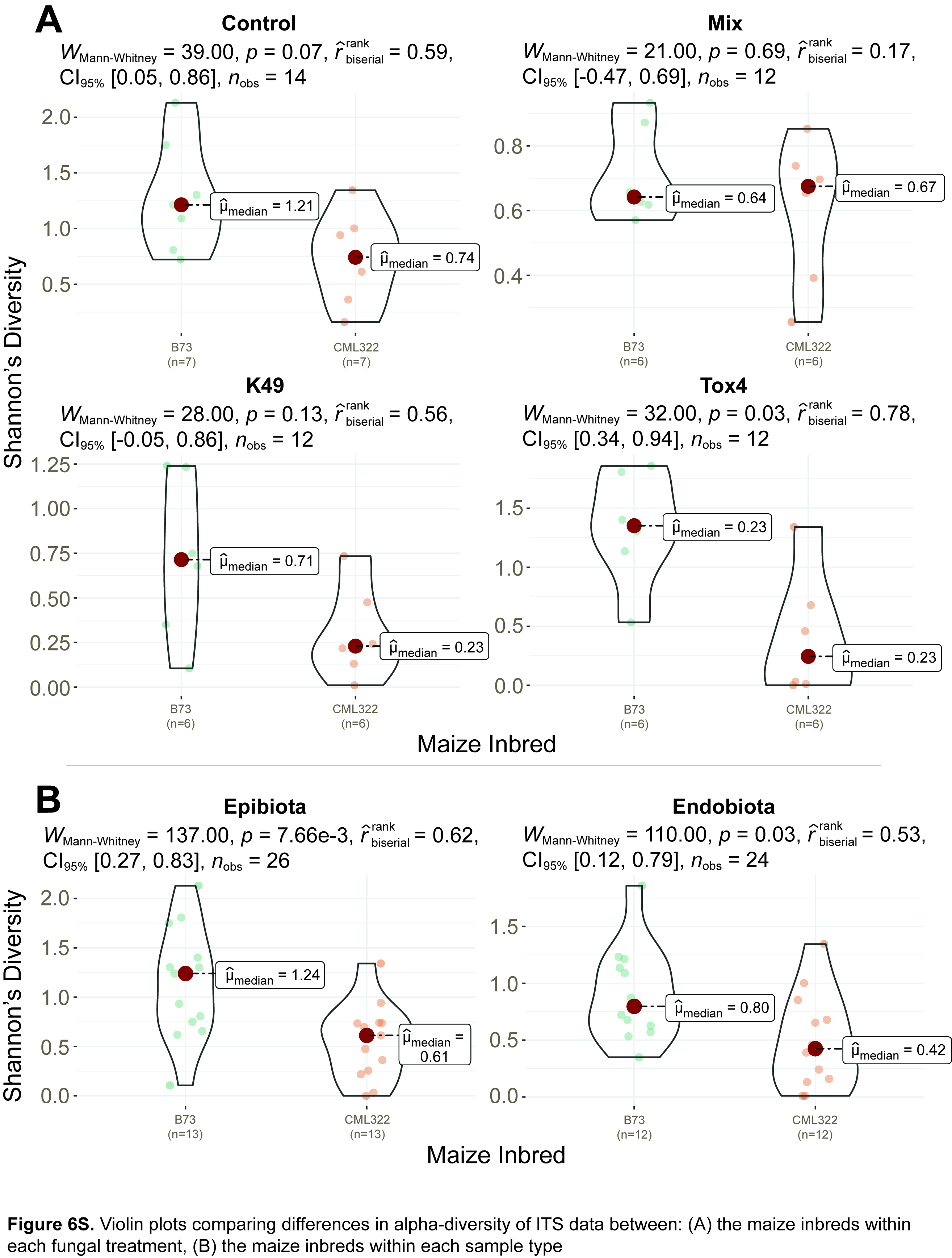

Supplement: Supplementary file 11 [file Image_6.JPEG]

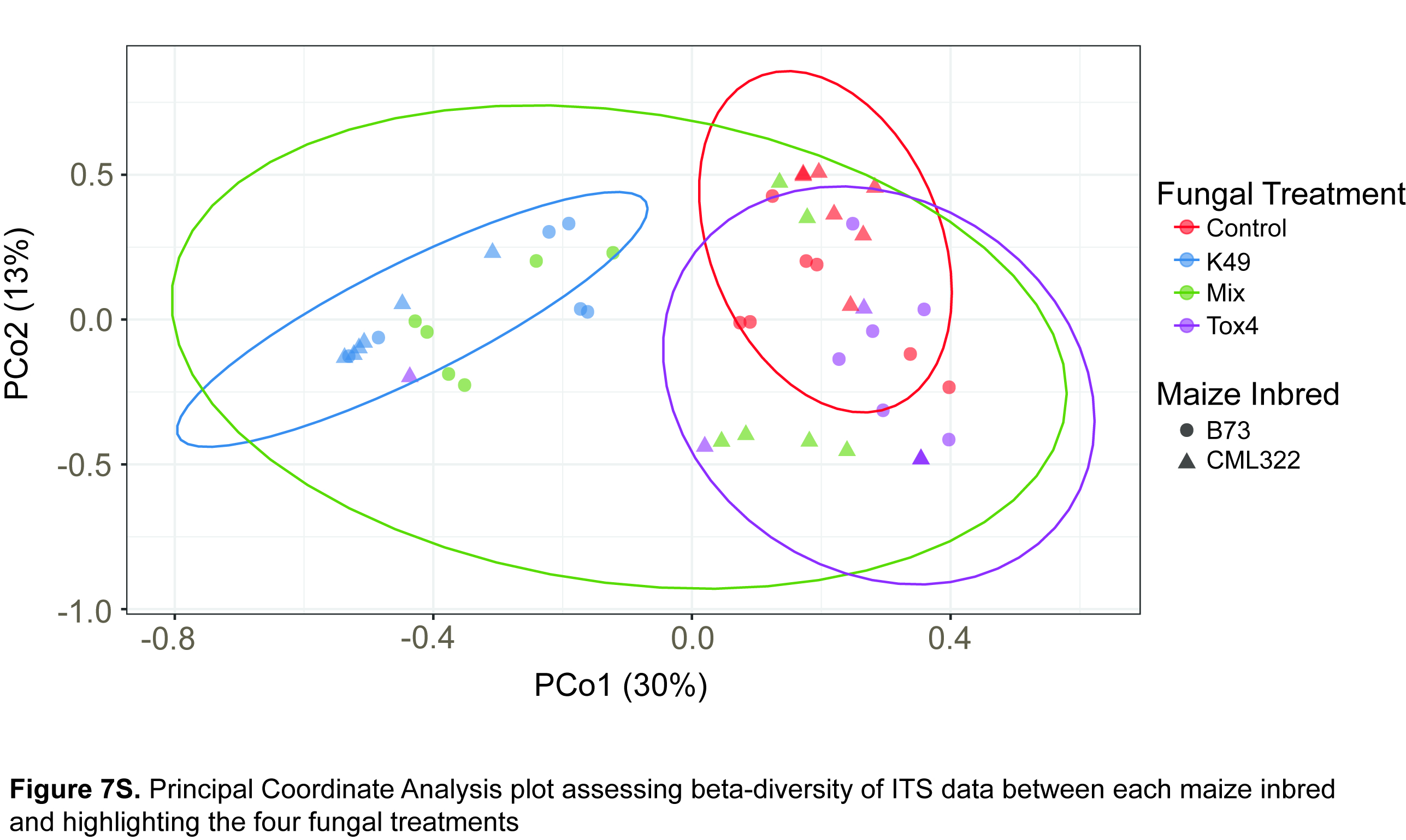

Supplement: Supplementary file 12 [file Image_7.JPEG]

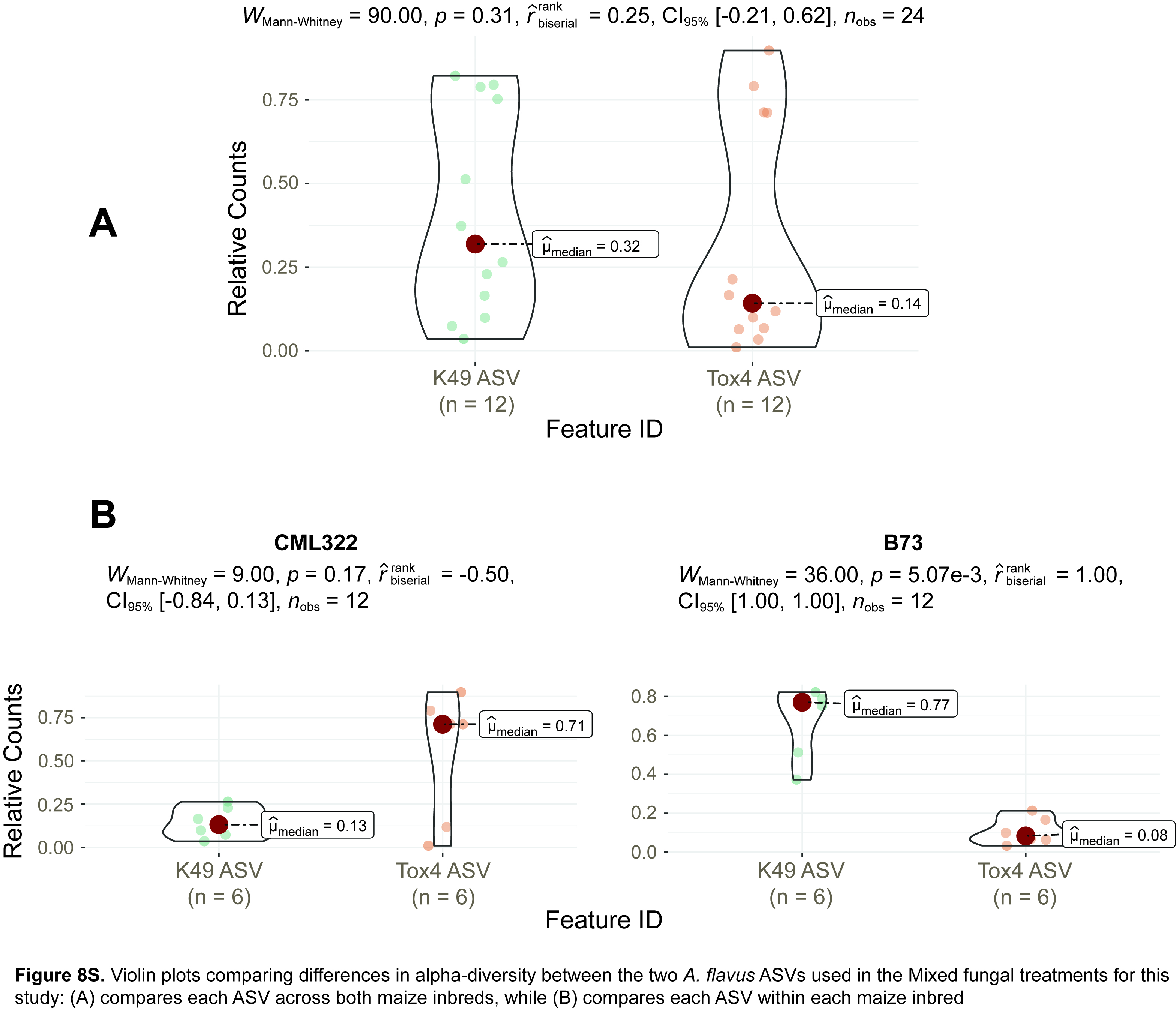

Supplement: Supplementary file 13 [file Image_8.JPEG]
